# Closely related methicillin-resistant *Staphylococcus aureus* isolates from retail meat, cows with mastitis, and humans in Japan

**DOI:** 10.1371/journal.pone.0187319

**Published:** 2017-10-30

**Authors:** Tomomi Sato, Masaru Usui, Noriko Konishi, Akemi Kai, Hidehito Matsui, Hideaki Hanaki, Yutaka Tamura

**Affiliations:** 1 Laboratory of Food Microbiology and Food Safety, Department of Health and Environmental Sciences, School of Veterinary Medicine, Rakuno Gakuen University, Ebetsu, Hokkaido, Japan; 2 Department of Microbiology, Tokyo Metropolitan Institute of Public Health, Shinjuku-ku, Tokyo, Japan; 3 Department of Microbiology, Tokyo Medical University, Shinjuku-ku, Tokyo, Japan; 4 Infection Control Research Center, Kitasato University, Minato-ku, Tokyo, Japan; Rockefeller University, UNITED STATES

## Abstract

Methicillin-resistant *Staphylococcus aureus* (MRSA) is a pervasive healthcare-acquired (HA) pathogen with recent emergence as a community-acquired (CA) pathogen. To elucidate whether meat mediates MRSA transmission between animals and humans in Japan, this study examined MRSA isolates from retail meat (n = 8), cows with mastitis (n = 7), and humans (HA-MRSA = 46 and CA-MRSA = 54) by molecular typing, virulence gene analyses, and antimicrobial susceptibility testing. MRSA isolates from retail meat were classified into sequence type (ST) 8/*spa* type t1767 (n = 4), ST8/t4133 (n = 1), ST59/t3385 (n = 1), ST88/t375 (n = 1), and ST509/t375 (n = 1). All seven MRSA isolates from cows with mastitis were ST8/t1767. 46 HA-MRSA were clonal complex (CC) 5, divided into t002 (n = 30), t045 (n = 12), and t7455 (n = 4). 54 CA-MRSA were classified into 6 different CCs: CC1 (n = 14), CC5 (n = 7), CC8 (n = 29), CC45 (n = 1), CC89 (n = 1), CC509 (n = 1), and into 16 different *spa* types including newly identified t17177, t17193, and t17194. The majority were CC8/t1767 (n = 16). CC of one CA-MRSA isolate (*spa* type t1767) was not classified. Among 41 CC8 MRSA (five from meat, seven from cows with mastitis, and 29 CA-MRSA), 14 ST8/SCC*mec* IVl isolates (three from meat, one from a cow with mastitis, and 10 CA-MRSA) had identical pulsed-field gel electrophoresis patterns and similar *spa* type (t1767, t4133, and t17177), and were typed as CA-MRSA/J (ST8/SCC*mec* IVl, positive for *sec* + *sel* + *tst* but negative for Panton–Valentine leukocidin and the arginine catabolic mobile element). These results suggest that there is a transmission cycle of CA-MRSA/J among meat, cows, and humans in Japan, although it is unclear whether the origin is cow.

## Introduction

*Staphylococcus aureus* is an important pathogen that causes infections in humans and is also responsible for disease in animals, such as bovine mastitis [1]. Treatment of infections caused by *S*. *aureus* has been further complicated by antimicrobial resistance in the bacteria, particularly methicillin-resistant *S*. *aureus* (MRSA) [2]. These infections were initially associated with hospitalization (healthcare-associated MRSA (HA-MRSA)). Recently, MRSA infections have been identified in healthy people living in the community, who had not been in hospital or had any medical treatment [2]. These cases are referred to as community-acquired MRSA (CA-MRSA) and represent a distinct group as compared to HA-MSRA.

The majority of typical HA-MRSA strains have the staphylococcal cassette chromosome *mec* (SCC*mec*) I, II, or III, and are classified into sequence type (ST), as defined by multi-locus sequence typing (MLST). ST5/SCC*mec* II known as the New York/Japan clone, ST22/SCC*mec* IV known as the epidemic MRSA (EMRSA)-15, and ST36/SCC*mec* II known as the EMRSA-16, are prevalent HA-MRSA linages [2]. CA-MRSA generally carries SCC*mec* type IV or V and often produces Panton–Valentine leucocidin (PVL), which causes tissue necrosis and leukocyte destruction [2]. ST8/SCC*mec* IVa (MRSA USA300), the most common CA-MRSA clone in the United States, is one of the most well-characterized CA-MRSA [3] and is now also a cause of hospital infection [4]. USA300 is positive for the arginine catabolic mobile element (ACME), which enhances colonization and survival in addition to PVL [3]. In the past decade, an increase in CA-MRSA belonging to clonal complex (CC)8, known as the CA-MRSA/J has been reported in Japan [5]; in contrast to USA300, CA-MRSA/J harbors SCC*mec* IVl and is negative for both PVL and ACME.

More recently, MRSA has been increasingly reported as emerging problem in the veterinary setting; MRSA has been isolated from cows, pigs, horses, and poultry worldwide (livestock-associated MRSA (LA-MRSA) [6]. LA-MRSA generally belongs to ST398 in European countries and ST9 in Asia [6]. It is largely associated with food producing animals but can colonize other host species, include causing infections in humans who are in frequent contact with MRSA-colonized pigs. However, about 20%– 40% of ST398 MRSA cases in humans cannot be epidemiologically linked to contact with livestock animals, indicating an alternative transmission pathway [7]. Additionally, ST398 MRSA also has been detected in retail food (veal, pork, and chicken meat) [8]. Previous studies have suggested that MRSA-positive pigs could contaminate the slaughterhouse environment, and have the potential to contaminate carcasses during the process [9], [10]. These results showed that MRSA are detected at different stages of the meat production chain and persist from farm to folk. The contamination of food products by animal MRSA is a big threat, as it has a potential for wide dissemination in the general population [11].

In Japan, MRSA have been detected in various food products, including chicken meat [12], [13], duck meat [13], meat products (the details regarding the type of meat product are unclear) [14], and bovine milk [15]. Furthermore, MRSA have been detected in livestock animals, including bovine mastitis [16] and nasal swabs of pigs [17], [18]. Although some MRSA related articles were reported in several origins (animals, meats, and humans), these MRSA isolates were not compared. Therefore, it has remained unsolved the relationship among these MRSA isolated from different origins. To elucidate the relationship among animal, meat, and human isolates, and to assess transmission from animals to humans in Japan, we investigated the characteristics of MRSA from retail meat, cows with mastitis, a common animal disease caused by *S*. *aureus*, and humans.

## Materials and methods

### Sample collection

A total of 5,435 food samples were collected from 2008 to 2009, and eight MRSA were isolated from eight meat samples used in this study. Various types of food (e.g. fish, rice balls) were included in addition to meat in the 5,435 samples, although the number of meat samples was uncertain. Eight MRSA were isolated from retail meat which was purchased in Osaka (n = 5: two ground beef samples and one sample each of pork ribs, ground pork, and Taiwanese frozen duck loin) and Tokyo (n = 3: one sample each of pork ribs, ground beef, and chicken) from 2008 to 2009. All meat, with the exception of the Taiwanese frozen duck loin, was produced domestically. MRSA from meat was isolated using a 1:10 dilution emulsion of the meat sample in sterile phosphate buffer saline. A total of 0.5 ml of the emulsion was added Tryptic to 4.5 ml of Soy Broth (TSB: Becton Dickinson Japan, Tokyo, Japan) with 7.5% NaCl and incubated for 18 to 20 h at 37°C. A loopful of enrichment broth was spread on Mannitol Salt Agar with Egg Yolk (MSEY; Eiken Chemical, Tokyo, Japan) and incubated for 48 h at 37°C. The presumptive colonies of *S*. *aureus* (yellow colonies with halo) were streaked and purified onto Trypticase Soy Agar (TSA: Becton Dickinson Japan). Isolates from meat were confirmed to be *S*. *aureus* by using PS LATEX (Eiken Chemical). The PCR was performed to confirm of the presence of the *mecA* gene [19].

Seven MRSA were isolated from seven cows with mastitis in 2011, all bred at the same private farm in Hokkaido. We isolated bacteria from the milk, which were taken from the breast, and identified MRSA to detect the pathogen of the mastitis by request from the owner. The owner of the farm consented to use of the isolates in this study anonymously, including non-disclosure of the city of the farm. We did not perform any animal experiments or field studies in this study. This study also did not involve endangered or protected species. Therefore, the special permission in the authorities for this investigation was not necessary. Milk samples were streaked onto MRSA screening agar (cefoxitin containing Mannitol Salt Agar with Egg Yolk (MS-CFX); Nissui Pharmaceutical, Tokyo, Japan) and overnight at 37°C. The presumptive colonies were further cultured onto TSA and repeatedly sub-cultured to get pure culture. Methicillin resistance was confirmed by testing for the presence of penicillin binding protein 2 (PBP2’) (MRSA-LA; Denka-Seiken, Tokyo, Japan).

A total of 100 human MRSA isolates collected in Kitasato University Hospital from 2014 to 2016 (46 HA-MRSA isolates and 54 CA-MRSA isolates) were obtained from the Infection Control Research Center, Kitasato University, Tokyo, Japan. All HA- and CA-MRSA isolates were recovered from blood samples. Infections were classified as either HA- or CA-MRSA according to origin of MRSA isolates and standard epidemiological definitions established by the U.S. Centers for Disease Control and Prevention [20]. MRSA isolates were classified as HA-MRSA if (i) they were isolated from a culture obtained 48 hours or more after a patient was hospitalized, (ii) the patient had a history of hospitalization, surgery, dialysis, or residence in a long-term care facility within 1 year before the MRSA culture date, (iii) the patient had an indwelling device at the time of culture, or (iv) the patient had a history of MRSA infection or colonization. All other MRSA isolates were considered CA-MRSA. We could not obtain information about the patients (age, symptoms, sex, and places of residence or infection) because of ethical constraints imposed by Kitasato University.

All MRSA isolates were confirmed to be *S*. *aureus* by matrix-assisted laser desorption/ionization time-of-flight mass spectrometry using the Bruker MALDI Biotyper system (Bruker Daltonics, Bremen, Germany) with the ethanol-formic acid extraction method. All were subsequently confirmed as *mecA*-positive by PCR [19].

### Molecular typing

For all MRSA isolates, SCC*mec* typing, phage open reading frame (ORF) typing and *spa* typing were performed. SCC*mec* typing was performed by multiplex PCR as described previously [21]. SCC*mec* types I to V were determined based on the *mec* complex class (*mec* classes A, B, and C) and the type of *ccr* (*ccr* 1, 2, 3, and 5) [21]. For MRSA isolates identified as SCC*mec*IV, further PCR for detection of the CWASP/J gene (*spj*) was performed to identify subtype SCC*mec* IVl [5]. The Clonal complex (CC) of all MRSA isolates were classified by phage ORF typing according to the methods described by Suzuki et al.[22]. Briefly, PCR was performed for the presence of 16 small genomic islets and scored according to islet presence (= 1) or absence (= 0). These scores were then converted to hexadecimal numbers using the internal bin2hex (number, places) function of Microsoft Excel, and the islet pattern (IP) was detected. In many cases, a one-to-one correspondence between the IP and CC identified by multilocus sequence typing (MLST) was observed. The IPs were compared with those previously reported by Suzuki et al.[22], and the CCs of MRSA isolates were classified. *spa* typing was performed as described previously [23].

Pulsed-field gel electrophoresis (PFGE) was performed for CC8 MRSA isolates with genetic DNA fragments generated using 30 U *Sma*I (TaKaRa, Otsu, Japan) as previously described [24]. Cluster analysis was performed with the software program BioNumerics v6 (Applied Maths, Sint-Martens-Latem, Belgium) using the Dice coefficient and the unweighted pair group method. MLST for retail meat, cows with mastitis, and CC8 human MRSA isolates was performed as described previously [25]. The founder and CC of each ST were determined using the enhanced version of Based Upon Related Sequence Types (eBURST) [26].

### Virulence gene analysis

The presence of genes encoding six staphylococcal enterotoxins, SEA to SEE, which main source of food poisoning [27], in addition to SEL, which CA-MRSA/J carries in Japan frequently [5] (SEs: *sea*, *seb*, *sec*, *sed*, *see*, and *sel*), toxic shock syndrome toxin-1 (TSST-1: *tst*) [27], which cause of TSS, exfoliative toxin A (ETA: *eta*) and B (ETB: *etb*), which are implicated in the cause of staphylococcal scalded-skin syndrome [27], PVL (*pvl*), which is associated with increased disease severity and found in a high proportion of CA-MRSA strains [19], and ACME (*acr*) which is a striking feature of USA300 and plays an important role in its growth and survival [3] was determined by PCR using previously reported primers [3], [19][27][28].

### Antimicrobial susceptibility testing

Antimicrobial susceptibility was tested by the agar dilution method following Clinical and Laboratory Standards Institute (CLSI) recommendations [29] for the following antibiotics: ampicillin (AMP; Sigma-Aldrich, St. Louis, MO, USA), oxacillin (OXA; Sigma-Aldrich), kanamycin (KAN; Sigma-Aldrich), gentamicin (GEN; Sigma-Aldrich), erythromycin (ERY; Sigma-Aldrich), clindamycin (CLI; Sigma-Aldrich), vancomycin (VAN; Sigma-Aldrich), ciprofloxacin (CIP; Sigma-Aldrich), and tetracycline (TET; Wako Pure Chemical Industries, Osaka, Japan). *S*. *aureus* ATCC 29213 and *Enterococcus faecalis* ATCC 29212 served as quality control strains. The breakpoints of these antimicrobial agents were determined according to CLSI interpretation criteria [29].

## Results

### Molecular characterization of MRSA isolates from meat, cows with mastitis, and humans

Characteristics of MRSA isolates in this study are summarized in Table 1. Among eight MRSA isolates from meat, two (one from ground pork and one from ground beef) were classified as ST8 (CC8)/t1767/SCC*mec* IVl, two (one from pork ribs and one from chicken) were ST8 (CC8)/t1767/SCC*mec* untypable (harbored *ccr* type 2, but multiplex PCR for *mec* class was not amplified), one from ground beef was ST8 (CC8)/t4133/SCC*mec* IVl, one from pork rib was ST88 (CC88)/t1028/SCC*mec* IV, one from ground beef was ST59 (CC59)/t3385/SCC*mec* V, and one from Taiwanese frozen duck loin was ST573/t3525/SCC*mec* IV (Table 1). All seven MRSA isolates from cows with mastitis were classified as ST8 (CC8)/t1767/SCC*mec* IVl. Among MRSA isolates from humans, all 46 HA-MRSA isolates were classified as CC5/SCC*mec* II, and were divided into *spa* type t002 (n = 30), t045 (= 12), and t7455 (n = 4). Fifty-four CA-MRSA isolates yielded 16 different *spa* types. These 16 *spa* types belonged to 6 different CCs: 14 of CC1 (t1784: n = 13, t2207: n = 1); 7 of CC5 (t002: n = 5, t045: n = 1, and newly identified t17193: n = 1), 29 of CC8 (t008: n = 2, t986: n = 1, t1476: n = 1, t1767: n = 16, t1852: n = 3, t 4133: n = 1, t12760: n = 1, and newly identified t17177: n = 3 and t17194: n = 1); one of each CC45 (t065), CC89 (t375), and CC509 (t375). The majority of CA-MRSA was CC8/t1767/SCC*mec* IVl (n = 15), following CC1/t1784/SCC*mec* IV (n = 12). SCC*mec* type of CC45 and CC89 were untypable (harbored *mec* class A, but multiplex PCR for *ccr* type was not amplified). CC of one CA-MRSA isolate, *spa* type t1767, was not able to be classified by phage ORF typing because its IP (04C6) was not reported previously. Accordingly, MLST was performed; however, ST was not able to identified because two of seven genes (*aroE* and *glpF*) could not amplify using primers described previously [25].

**Table 1:** Molecular characterization of MRSA isolates from meat, cow mastitis, and humans (HA-MRSA and CA-MRSA).

CC^a^	SCC^b^ *mec*	*spa*	Origin				Resistant isolates									Pattern of virulence genes
			Meat (n = 8)	Cow with mastitis (n = 7)	CA-MRSA (n = 54)	HA-MRSA (n = 46)	AMP^f^	OXA	KAN	GEN	ERY	CLI	VAN	CIP	TET	
**1**	IV	t1784			13		13	13	1	1	13	0	0	13	0	*sea* (n = 9), *sea* + *see* (n = 3), negative (n = 1)
		t2207			1		1	1	0	0	1	0	0	1	0	negative
**5**	II	t002			4	30	34	34	32	26	34	33	0	34	27	*seb* (n = 13), *sec + sel + tst* (n = 11), *sea + sec + sel + tst* (n = 2), *sel + tst* (n = 1), *sel* (n = 1), negative (n = 6)
		t045			1	12	13	13	13	7	13	13	0	10	13	*sec + sel + tst* (n = 10), *seb + sec + sel + tst* (n = 2), *sea + sec + see + sel + tst* (n = 1)
		t7455				4	4	4	4	3	4	4	0	4	4	*sec + sel + tst* (n = 3), *seb + sec + sel + tst* (n = 1)
	IV	t002			1		1	1	0	0	0	0	0	0	0	*tst* (n = 1)
		t17193			1		1	1	0	0	1	0	0	0	0	negative
**8**	IV	t008			2		2	2	2	0	2	0	0	1	0	*pvl + acr* (n = 2)
		t986			1		1	1	1	1	1	0	0	1	0	negative
		t1476			1		1	1	0	0	0	0	0	0	0	*tst*
		t1767			1		1	1	0	0	0	0	0	0	0	*sel* + *tst*
		t1852			3		3	3	3	3	0	0	0	3	0	negative (n = 3)
		t17194			1		1	1	0	0	1	0	0	1	0	negative
	IVl	t1767	2 (1 GB, 1 GP)^e^	7	15		24	24	24	19	9	0	0	0	0	*sec* + *sel* + *tst* (n = 23), negative (n = 1)
		t4133	1 (GB)		1		2	2	2	2	0	0	0	0	0	*sec + sel + tst* (n = 1), negative (n = 1)
		t17177			3		3	3	3	3	0	0	0	0	0	*sec + sel + tst* (n = 3)
	V	t12760			1		1	1	1	1	1	0	0	1	0	negative (n = 1)
	UT^c^	t1767	2 (1 PR, 1 C)				2	2	2	2	0	0	0	0	0	*sec + sel + tst* (n = 2)
**45**	UT^d^	t065			1		1	1	0	0	0	0	0	0	0	negative
**59**	V	t3385	1 (GB)				1	1	0	0	0	0	0	0	0	*sea + seb + sel*
**88**	IV	t1028	1 (PR)				1	1	1	0	0	0	0	0	0	negative
**89**	UT^d^	t375			1		1	1	1	1	1	1	0	0	0	*etb*
**509**	II	t375			1		1	1	1	1	1	1	0	0	0	negative
**573**	IV	t3525	1 (TD)				1	1	1	1	0	0	0	0	1	*sec*
**Unclassfied**	IV	t1767			1		1	1	0	0	0	0	0	0	0	*sed*

^a^; The Clonal complex (CC) of all MRSA isolates were classified by phage ORF typing

^b^; SCC*mec* types I to V were determined based on the *mec* complex class (*mec* classes A, B, and C) and the type of *ccr* (*ccr* 1, 2, 3, and 5)

^c^; Untypable (*ccr* type 2 + *mec*-untypable)

^d^; Untypable (*ccr*-untyable + *mec* class A)

^e^; GB: ground beef, GP: ground pork, PR: pork ribs, C: chicken, TD: Taiwanese frozen duck loin

^f^; AMP: ampicillin, OXA: oxacillin, KAN: kanamycin, GEN: gentamicin, ERY: erythromycin, CLI: clindamycin, VAN: vancomycin, CIP: ciprofloxacin, TET: tetracycline

### Toxin genes of MRSA isolates from meat, cows with mastitis, and humans

The toxin genes detected in each MRSA isolate are summarized in Table 1. In total, 88% (7/8) from meat, 100% (7/7) from cows with mastitis, 93% (43/46) HA-MRSA isolates, and 70% (38/54) of CA-MRSA isolates carried at least one toxin gene. Among the fourteen CC1/SCC*mec* IV MRSA isolates, *sea* (n = 9) was most common, followed by *sea* + *see* (n = 3). Among the 51 CC5/SCC*mec* II MRSA isolates, including 46 HA-MRSA and 5 CA-MRSA isolate, *sec* + *sel* + *tst* (n = 24) was most common, followed by *seb* (n = 13), *seb* + *sec* + *sel* + *tst* (n = 3), *sea* + *sec* + *sel* + *tst* (n = 2), *sea* + *sec* + *see* + *sel* + *tst* (n = 1), *sel* + *tst* (n = 1), and *sel* (n = 1). One CC5/SCC*mec* IV carried *tst*. Among the nine CC8/SCC*mec* IV MRSA isolates, *pvl* + *acr* (n = 2), *sel* + *tst* (n = 1), and *tst* (n = 1) were observed. Among the twenty-nine CC8/SCC*mec* IVl MRSA isolates, including three from meat, seven cows with mastitis, and nineteen from humans, 97% (27/29) carried *sec* + *sel* + *tst*. Two CC8 SCC*mec* untypable isolates from pork ribs and chicken carried *sec* + *sel* + *tst*. One CC59/SCC*mec* V (isolated from ground beef) carried *sea + seb + sel*, one CC573/SCC*mec* IV (isolated from Taiwanese frozen duck) carried *sec*, and one CC unclassified/SCC*mec* IV (belonging to CA-MRSA) carried *sed*.

### Antimicrobial susceptibility

All MRSA isolates in this study were resistant to β-lactams (AMP and OXA); however, they were susceptible to VAN. In addition, CC8/SCC*mec* IVl was resistant to KAN (29/29: 100%) and GEN (24/29: 83%). CC5/SCC*mec* II was resistant to all tested antimicrobial agents except for VAN.

### PFGE analysis and MLST of CC8 MRSA isolates

**Fig 1:**
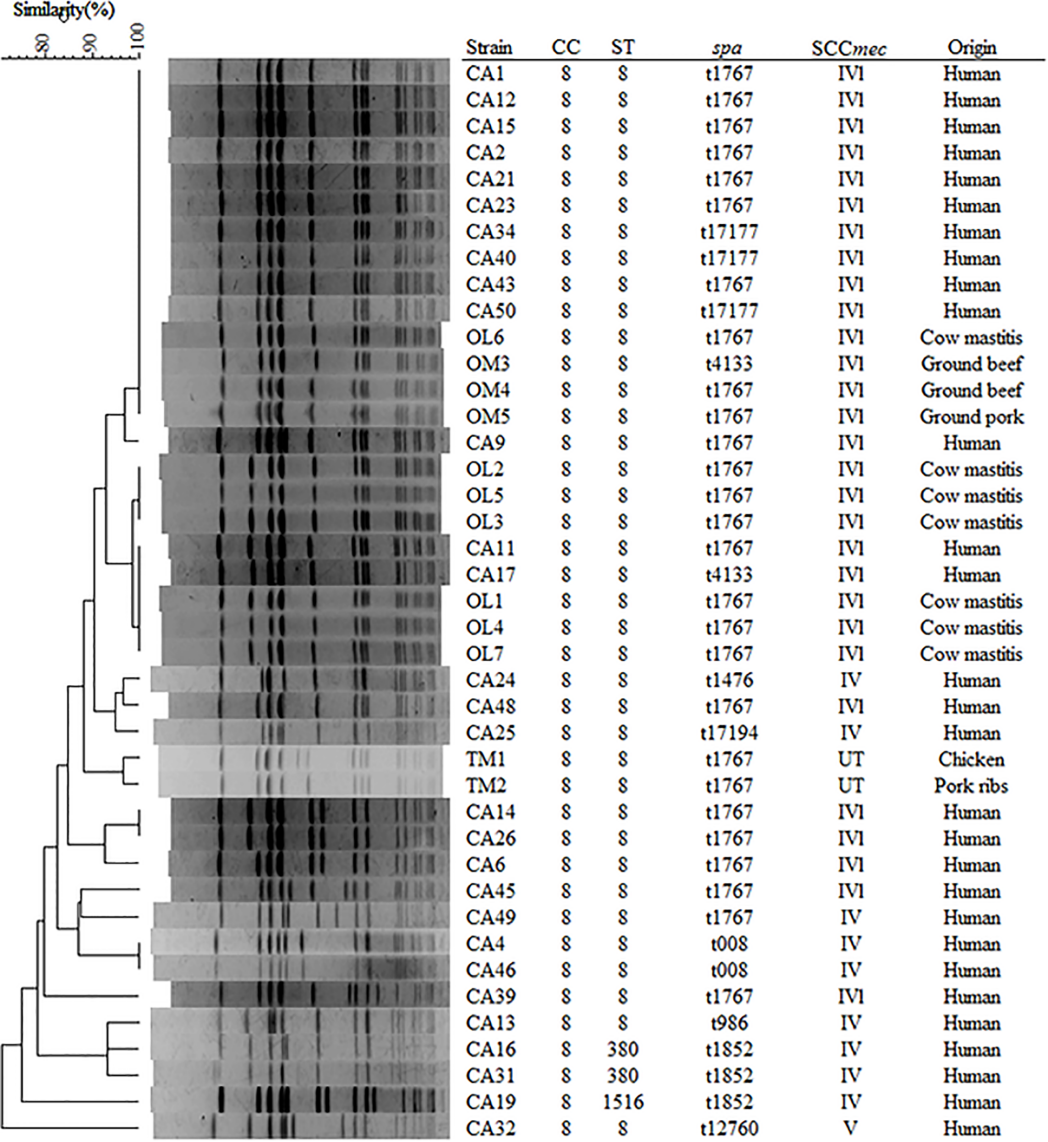
Genetic relationships among CC8 MRSA isolates. UPGMA dendrogram showing genetic relatedness among representative CC8 MRSA isolates as determined by PFGE with *SmaI*. UT; *ccr* type 2 + *mec*-untypable.

PFGE analysis and MLST were performed to classify MRSA isolates according to CC8 (n = 41, determined by phage ORF typing), which was the common clone among retail meat (n = 5), cows with mastitis (n = 7), and CA-MRSA from humans (n = 29) (Fig 1). CC8 isolates were classified into a total of three STs: ST8 (allelic profile 3-3-1-1-4-4-3), ST380 (3-3-61-42-4-4-3), and ST1516 (3-3-1-42-4-4-3). Three CC8/SCC*mec* IVl MRSA isolates from meat (two from beef and one from pork), one from a cow with mastitis, and ten human CA-MRSA isolates showed 100% PFGE similarity. Similarly, two human CA-MRSA isolates and three from cow with mastitis showed 100% similarity with different origin. These were ST8/SCC*mec* IVl containing three *spa* types with similar repeat profiles (t1767: 11-19-12-21-17-34-24-24-34-22-25, t4133: 11-12-21-17-34-24-24-34-22-25, and t17177: 11-19-12-21-17-34-24-24-24-24-34-22-25).

## Discussion

This study showed that three MRSA isolates from retail meat, one MRSA from a cow with mastitis, and ten CA-MRSA isolates were closely related according to *spa t*ype, and identical according to PFGE pattern, ST, and SCC*mec*, they all had the characteristics of ST8/SCC*mec* IVl. Our study is the first to detect the closel molecular epidemiological relationship of MRSA among retail meat, cows with mastitis, and CA-MRSA from humans in Japan.

ST8 MRSA isolated in this study showed a similar genotype and antimicrobial susceptibility pattern to ST8 CA-MRSA/J in Japan, which has some different characteristics from foreign countries. In Japan, a ST8 CA-MRSA/J strain was described; ST8 CA-MRSA/J which can be characterized as carrying SCC*mec* IVl, *spa* type t1767, negative for PVL and ACME, positive for *sec*, *sel*, and *tst*, and resistant to gentamicin [4]. It is reported that 37.5% (18/48) of CA-MRSA from human were typed as ST8 CA-MRSA/J in Japan [5]. Gentamicin is used in an outpatient for the treatment of skin infections in Japan [30]. Therefore, antibiotic therapy based on antimicrobial susceptibility test is needed for skin infections caused by MRSA. ST8/SCC*mec* IVa, positive for PVL and ACME MRSA (USA300), is a predominant CA-MRSA genotype in the US and worldwide [31]. USA300 clones show resistance to many non-β-lactams (macrolides, fluoroquinolones, and tetracycline) in addition to β-lactams [31]. ST8 MRSA isolates used in this study showed the same genotype and antimicrobial profile, aminoglycosides resistance, as those of CA-MRSA/J. Although the geographical area where human MRSA was derived, as well as the sample size of meat and animals were all limited, this study revealed that ST8 CA-MRSA/J spreads not only to the human community setting, but also among meat and living livestock (cow), but not yet to the healthcare setting.

Four STs (ST8, ST59, ST88, and ST573) were identified in isolates from retail meat in this study. All of these STs are human-associated types: ST8 in the United States and worldwide [31], ST59 in Taiwan [31], ST88 in Africa and Asia [32], ST573 which is a rare clone found previously in Taiwan [33] and Australia [34]. Three of four STs (ST8, ST59, and ST88) were found primarily in human CA-MRSA in Japan [2]. MRSA isolates from retail chicken and duck meat in Japan were ST8/SCC*mec* IV, and regarded as CA-MRSA [13]. These observations suggest a relationship of MRSA between retail meats and humans. However, these four STs have not been reported in Japanese livestock animals, and the prevalence of MRSA is low [0.9%–8% [17][18]] in Japanese livestock. Considering these reports, there is a strong possibility that MRSA isolates from meat in this study are contaminated from humans, although we cannot draw any definitive conclusions regarding the source of contaminated retail meat with MRSA.

Among STs from retail meat, ST573 MRSA from Taiwanese duck loin was first isolated in Japan. ST573 is a rare clone, found previously in Taiwanese children [SCC*mec* V, 0.3% (1/294)] [35], healthcare settings [SCC*mec* IV, 2.4% (5/206)] in Taiwan [33], and in community settings [SCC*mec* V, 0.05% (2/4,099)] in Australia [34]. There are several reports of pathogens associated with the import of food; an oxacillin-susceptible *mecA*-positive *S*. *aureus* (OS-MRSA), has never been isolated in Europe, was isolated from imported cheese. It might indicate that OS-MRSA may enter the EU via the import of food [36]. Although the source of ST573/SCC*mec* IV MRSA from Taiwanese frozen duck in this study is unclear, it might have been brought to Japan via imported meat from Taiwan, the only region where ST573 carrying SCC*mec* IV MRSA has been detected [33].

Since *S*. *aureus* can produce enterotoxins, it also poses a threat to humans who ingest food contaminated with these toxins [37]. Staphylococcal food poisoning, characterized by vomiting and diarrhea, is a leading cause of food-borne illness in Japan [38]. Food sources of *S*. *aureus* have expanded to include livestock animal products and low-fat milk [38]. Toxic shock syndrome (TSS), which can be life-threatening, is defined by clinical and laboratory evidence of fever, rash, desquamation, hypotension, and multiple organ failure caused not only by toxic shock syndrome toxin-1 (TSST-1), but also by enterotoxins [39]. In this study, 88% (7/8) of MRSA isolates from retail meat were positive for enterotoxin genes and *tst*, higher than previous study [28.6% [13]]. In Japan, the contamination rate of MRSA in meat is low [0.45% to 1.5% [12][13]], but MRSA isolates from retail meat frequently carry virulence genes, and the spread of MRSA can cause human disease via the handling of contaminated retail meat.

## Conclusion

This study showed that ST8 CA-MRSA/J is detected in the community setting, including retail meat and cows, and suggested that there is the transmission route of ST8 CA-MRSA/J among these sources. However, the direction of transfer of MRSA could not be established, and the results might not be reflective of Japan overall because the number of MRSA isolates from meat and animals was very low. Additional studies are needed to determine the origin of MRSA from retail meat, confirm the distribution of ST8 CA-MRSA/J in living animals, and assess the risk of the spread of MRSA to consumers and others who handle meat.

## Supporting information

S1 Table Characteristics of MRSA isolates in this study. (XLSX)
